# Observing errors in a combination of error and correct models favors observational motor learning

**DOI:** 10.1186/s12868-021-00685-6

**Published:** 2022-01-04

**Authors:** Zhi-Ming Tang, Yutaka Oouchida, Meng-Xin Wang, Zu-Lin Dou, Shin-Ichi Izumi

**Affiliations:** 1 Department of Rehabilitation Medicine, Yuedong Hospital, The Third Affiliated Hospital of Sun Yat-sen University, Meizhou, 514000 China; 2 Department of Rehabilitation Medicine, The Third Affiliated Hospital of Sun Yat-Sen University, Guangzhou, 510630 China; 3 Department of Physical Medicine and Rehabilitation, Tohoku University Graduate School of Medicine, Sendai, 980-8575 Japan; 4 Department of Physical Medicine and Rehabilitation, Tohoku University Graduate School of Biomedical Engineering, Sendai, 980-8575 Japan; 5 Department of Education, Osaka Kyoiku University, Osaka, 582-8582 Japan

**Keywords:** Observing errors, Imitative learning, Motor learning, Observation, Mixed model

## Abstract

**Background:**

Imitative learning is highly effective from infancy to old age; however, little is known about the effects of observing errors during imitative learning. This study aimed to examine how observing errors affected imitative learning performance to maximize its effect.

**Methods:**

In the pre-training session, participants were instructed to pinch at a target force (8 N) with auditory feedback regarding generated force while they watched videos of someone pinching a sponge at the target force. In the pre-test, participants pinched at the target force and did not view a model or receive auditory feedback. In Experiment 1, in the main training session, participants imitated models while they watched videos of pinching at either the incorrect force (error-mixed condition) or target force (correct condition). Then, the exact force generated was measured without receiving auditory feedback or viewing a model. In Experiment 2, using the same procedures, newly recruited participants watched videos of pinching at incorrect forces (4 and 24 N) as the error condition and the correct force as the correct condition.

**Results:**

In Experiment 1, the average force was closer to the target force in the error-mixed condition than in the correct condition. In Experiment 2, the average force in the correct condition was closer to the target force than in the error condition.

**Conclusion:**

Our findings indicated that observing error actions combined with correct actions affected imitation motor learning positively as error actions contained information on things to avoid in the target action. It provides further information to enhance imitative learning in mixed conditions compared to that with correct action alone.

## Background

Imitation plays a central role in human motor skill learning [1, 2]. During imitation learning, learners observe the actions of others as a model, which provides them with important sources of information when acquiring motor skills [3]. Many studies have provided evidence that observing another person performing an action activates the same sensorimotor representation as the observed action [4, 5], which reflects mental simulation of the observed action in the mirror neuron system [6–8]. According to these previous findings, this mental simulation of observed action plays an important role in acquiring a new motor skill in imitative learning [9, 10].

Which model should be used in imitation to maximize learning? Previous studies have found that observing a model performing a skilled action can enhance motor learning [11, 12]. A skilled model can provide many motor parameters of action for performing tasks or useful movement strategies, presumably enabling the observer to form a “perceptual blueprint” of the task to be learned [13]. Another explanation is the direct matching hypothesis that perceived movements automatically activate existing internal motor components in the same way as actual movements [14, 15]. According to this hypothesis, imitative motor learning could be facilitated if the observed action was the same (i.e., a correct model) as the target action to be learned [15]. Previous behavioral experiments have detected this facilitation effect during movement performance when the observed movement was congruent with the targeted movement [16]. Therefore, it is natural that in imitative learning, the same or similar movements are used as a model to maximize the effect of imitative learning. Additionally, using the first-person perspective in imitation learning is considered more effective than the third-person perspective as an accurate state estimation for actions is perceived, which map more closely onto visual input of self-generated action [17].

Arguably, an important characteristic of being “skilled” is the capacity not only to perform an action accurately as planned but also to detect differences between the planned and executed actions, known as errors, and correct them [3, 18]. The feedback error learning model considers that when a movement is made, the sensorimotor system receives its outcome and compares it with a desired or predicted outcome [3, 19]. The difference between the movement’s outcome and desired outcome is known as a self-generated error. While acquiring a new motor skill, this self-generated error is calculated and corrected in every movement to eliminate discrepancies. Owing to mental simulation by the mirror neuron system, only an observation of the other’s action can trigger the calculation and modification of the error, thereby leading to observational learning without physical movement [20].

Compared to observing a skilled model, inconsistent effects of observing novice models have been reported. Pollock, using a computer tracking game, has found that observing unskilled trials has the same effect on motor learning [21]. In Moore et al.’s study, the subjects observe unskilled and skilled models, but no differences are reported in the learning outcomes [22]. Some studies have reported that the learning effect when both novice and skilled models are observed is larger than when only skilled models are observed in the same number of trials [1, 2, 12, 23]. However, observing novice models alone does not have the same magnitude of effect as observing skilled models. The difference in learning strategy may be because, when observing a skilled model, the attention focus is directed at pattern analysis, while novice models support attention focus being allocated to strategy identification first [11].

Thus, the results of previous studies suggest that learners could benefit not only from a skilled model but also from a novice model exhibiting poor performance, probably by compensating for the gap between the desired performance and the errors in the novice performance. However, the novice model in the previous study is different from the error model as the former intends to achieve the goal, even thought he did not reach the goal because the poor process [12]. It is thus unclear from the previous studies how error information affects imitative motor learning and how observing both the error and correct model affects learning performance in imitative learning.

In this study, we evaluated the effect of observing error actions included in correct models when learning to pinch an object at the target force by imitative learning.

## Materials and method

### Participants

A total of 50 undergraduate students participated in the study. There were 30 participants (aged 28.5 ± 6.6 years; 13 females, four left-handed) in Experiment 1 and 20 (aged 27.5 ± 3.0 years; 10 females, all right-handed) in Experiment 2. All participants were screened to rule out medication use, a history of neurological or psychiatric disorders, head trauma, substance abuse, or other serious medical conditions. The study was approved by the Ethics Committee of the Medical Faculty of Tohoku University. Written informed consent was obtained from all the participants before the study.

### Apparatus and video stimuli

**Fig. 1:**
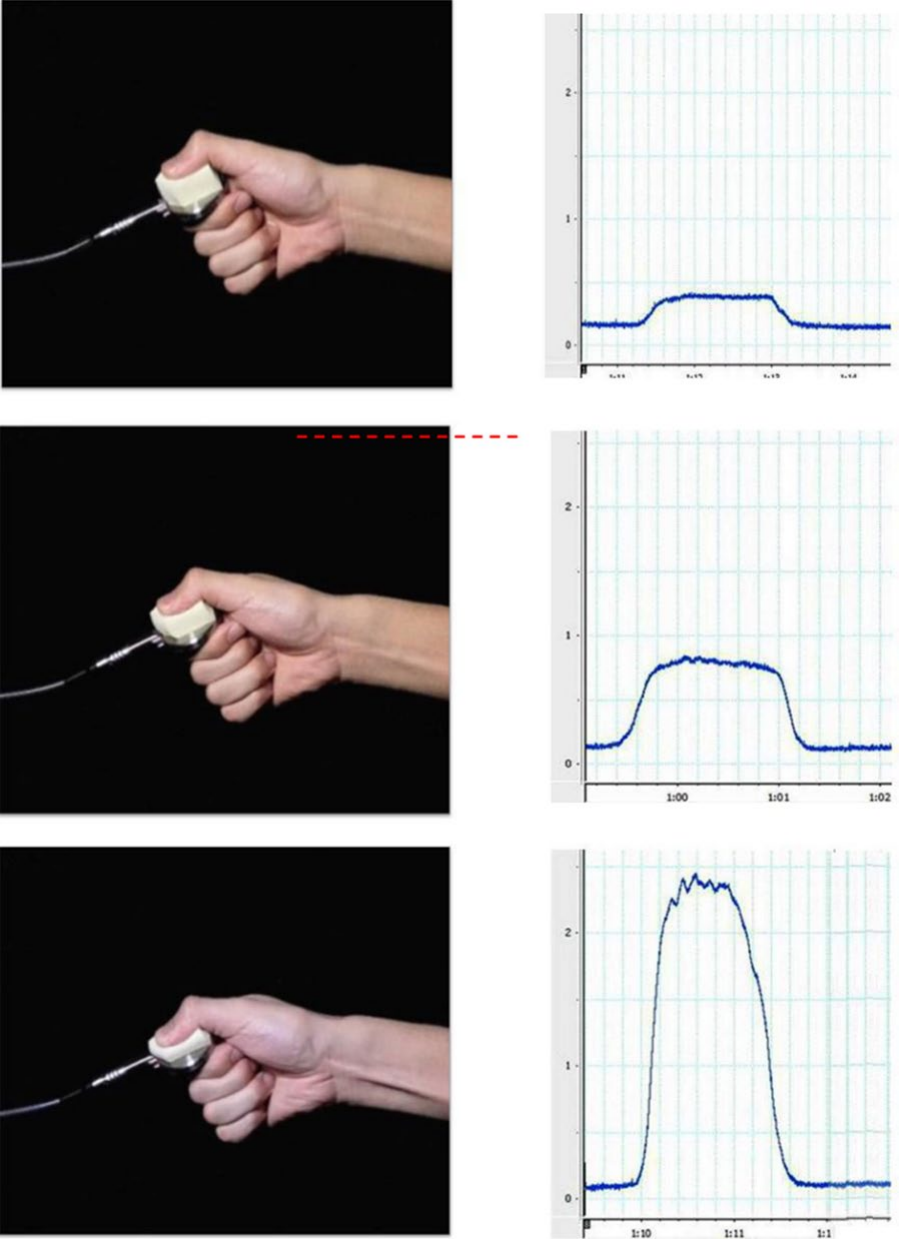
Three types of observational pinching actions. The images show the modal compaction point of each type of pinching action video. The wave on the right-side image shows the force change during the pinching action on the left side. Actions displayed in the video: upper, 4 N; middle, 8 N; down, 24 N. The force of the 8 N action was used as the target model, while the other actions were used as error models

As shown in Fig. 1, a custom-made pinching device was used, which consisted of a sponge (Ishihara Co. Ltd, Japan) and a pinching sensor (H500 Hand Kit; Biometrics Ltd, UK). The pinching device was connected to an E-Link (Biometrics Ltd., UK) that amplified the signal from the force sensor to a Powerlab (Adinstruments Ltd., USA) to transform analog data into digital data. The Powerlab system was used to measure the peak force in each trial using the LabChart software. A 15.6-inch notebook personal computer (Apple 107T5; refresh rate = 85 Hz) was used to present the visual stimuli to the participants. The experiment was programmed and controlled by a handmade software using MATLAB (Version 2010a; Math Works Inc., USA).

A total of three types of pinching action at different peak forces (4, 8, and 24 N) were performed by an experimenter and recorded with a high-speed camera (CASIO EX-F1, Max 1200 fps) to create the observational models. Each pinching action could easily be discriminated by the thickness of the sponge compressed by the pinching (see Fig. 1); that is, the compressed thickness under 4 N was almost 3/4 of the thickness of the original sponge, while 8 N was almost 1/2, and 24 N was almost 1/4. The participants were asked to pinch a force sensor for 3 s. The target force was 8 N in all conditions and was almost at 10–15% of the maximum voluntary contraction of pinching action in healthy adults, as assessed in the pre-experiment.

Participants sat on a chair in front of a table on which they placed their right or left hand, with their fingers in the pinching position. The distance from the participant’s eyes to the screen of the personal computer was 65 cm. The height of the chair was adjusted to make the participants comfortable.

### Procedures

The experiment consisted of four sessions: “pre-training,” “pre-test,” “main training,” and “post-test” (Table 1).

**Table 1:** Designs of Experiments 1 and 2

Session condition	Pre-training	Pre-test	Main training	Post-test
Error-mixed	Observe correct model with PP + KR (10 trials)	Perform PP without KR (20 trials)	Observe error-mixed model with PP + KR (20 trials × 5 blocks)	Perform PP without KR (20 trials)
Error			Observe Error model with PP + KR (20 trials × 5 blocks)	
Correct			Observe correct model with PP + KR (20 trials × 5 blocks)	

Experiment 1: participants underwent the error-mixed and correct conditions

Experiment 2: participants underwent the error and correct conditions

Error-mixed model: The observational actions consisted of three types of pinching actions with peak forces of 4, 8, and 24 N. The appearance rates of 4, 8, and 24 N were 25%, 50%, and 25%, respectively

Error model: The observational actions consisted of two types of pinching action with peak forces of 4 and 24 N. The appearance rates of 4 and 24 N were 50% each

Correct model: The observational action consisted of only one type of pinching action with a peak force of 8 N

*PP* Physical practice, *KR* knowledge of the results

There were three conditions that were different during the main training: error-mixed, correct only, and error only conditions. In the error-mixed condition, the observed action trials consisted of 25% of 4 N, 50% of 8 N, and 25% of 24 N. In the correct condition, only 8 N was used. In the error condition, 4 N and 24 N were 50% each.

**Fig. 2:**
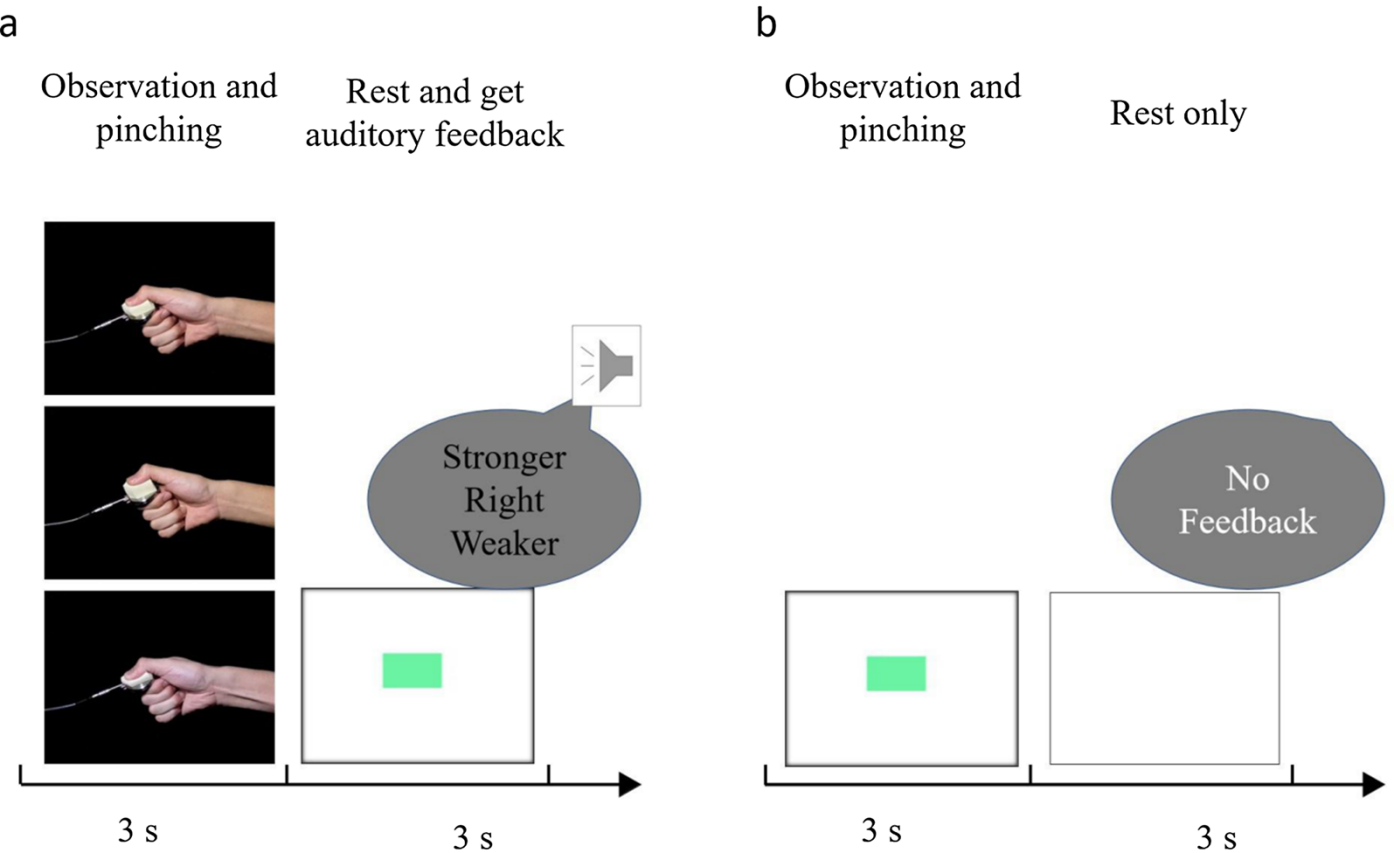
**a** The protocol used during pre-training and main training session. The participant was instructed to pinch with a peak force of 8 N while they watched the pinching action in the video. They received information regarding their results from auditory feedback when the green bar was shown on the screen. In the pre-training session of both the conditions and the main training session of the correct condition, the observed pinching action was 8 N only. In the main training session of the error-mixed condition, the observational actions were 4, 8, and 24 N, which were shown randomly. **b** Protocol used during pre-test and post-test session. Participants were instructed to pinch with a force of 8 N as they learn in the training session. No information regarding the results was provided

Before the experiment, we explained the experimental protocol to the participants in detail. Participants were evaluated several times to make sure they understood the method thoroughly. In the “pre-training” session, the participants were instructed to pinch a force sensor at the peak force of 8 N 10 times while observing the pinching action performed by an experimenter at 8 N on video. In both the “pre-training” and “main training” sessions, an experimenter provided auditory feedback regarding their pinching force, which was “stronger than the target force,” “right force,” or “weaker than the target force” (Fig. 2a).

In the “pre-test” and “post-test” sessions, participants were instructed to pinch at the peak force of 8 N 20 times without viewing a video or receiving feedback about their force (Fig. 2b), to measure the learning effect by comparing the pre- and post-test results.

The “main training” session had three conditions: correct model, error model, and error-mixed model conditions. The “main training” session comprised five blocks, with 20 trials in each block. In the correct model condition, participants were instructed to pinch at 8 N 100 times while observing pinching actions at the peak force of only 8 N. In the error model condition, participants were instructed to pinch while receiving auditory feedback regarding their force while observing pinching at 4 N or 24 N (each force video appeared 50% of the time). In the error-mixed model condition, they pinched while randomly observing pinching at a peak force of 4, 8, or 24 N (see Fig. 2a). The correct range of peak force was from 7.6 N to 8.4 N, which was 8 N ± 5% × 8 N.

There were two minutes between the sessions, and one minute between the blocks. All participants pinched using their left and right hands based on whether they took part in Experiment 1 or 2, and counterbalancing was conducted between handedness (dominant and non-dominant) and the conditions (error-mixed and correct in Experiment 1; error and correct in Experiment 2).

#### Experiment 1

In Experiment 1, error-mixed and correct conditions were given to the participants in the main training session.

#### Experiment 2

In Experiment 2, the participants were given the error and correct conditions, as described in Table 1. The experimental setting was the same as in Experiment 1, and pinching at error forces 4 N and 24 N appeared randomly at 50% rate.

### Data analysis

To measure the learning effect of the pinching force, we calculated the root mean square of the deviation between the peak and target forces (8 N). For each trial:

$$\mathrm{Deviation }= \sqrt{{(peakforce-8)}^{2}}(\mathrm{Unit}:\mathrm{ N})$$

The experimental design was a 2 (timing: pre and post) × 2 (model: either error-mixed in Experiment 1 or error model in Experiment 2 vs. correct model) repeated measures analysis of covariance (rm ANOVA). To analyze the learning curve in the main training session in both Experiments 1 and 2, we also conducted a 2 (models: either error-mixed in Experiment 1 or error in Experiment 2 and correct model) × 5 (times: first to fifth blocks in the main training session) rm ANOVA. In the post hoc test, multiple comparisons with Bonferroni correction were performed; the represented p value was the adjusted value. Data are shown as mean ± Standard error (SE). The significance level was set at *p* < 0.05.

## Results

### Experiment 1 (error-mixed model vs. correct model)

**Fig. 3:**
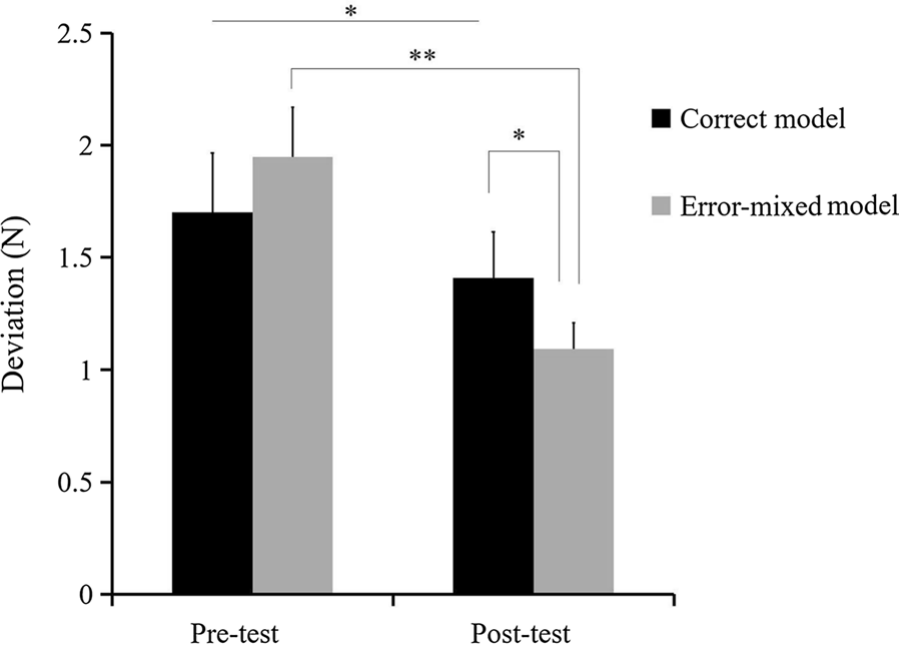
The deviation during the pre-test and post-test for each condition in Experiment 1. Error bars represent standardized error. *p < 0.05, **p < 0.01

The deviations of the two conditions (error-mixed and correct) and the two time levels (pre-test and post-test) are shown in Fig. 3. The main effect of time was significant [F (1, 29) = 20.10, *p* < 0.001], which showed that the participants experienced a learning effect after the main training session based on a comparison of the deviation of the pre-and-post tests. The main effect of condition was not significant [F (1, 29) = 0.070, *p* = 0.794]. A significant interaction was found between time and condition [F (1, 29) = 6.219, *p* = 0.019]. No significant difference was found between the correct (1.72 ± 0.26 N) and error-mixed conditions (1.95 ± 0.22 N) (*p* = 0.374) in the pre-test, while in the post-test, the deviation of the error-mixed condition (1.09 ± 0.11 N) was significantly lower than compared to the correct condition (1.41 ± 0.21 N) (*p* = 0.037).

**Fig. 4:**
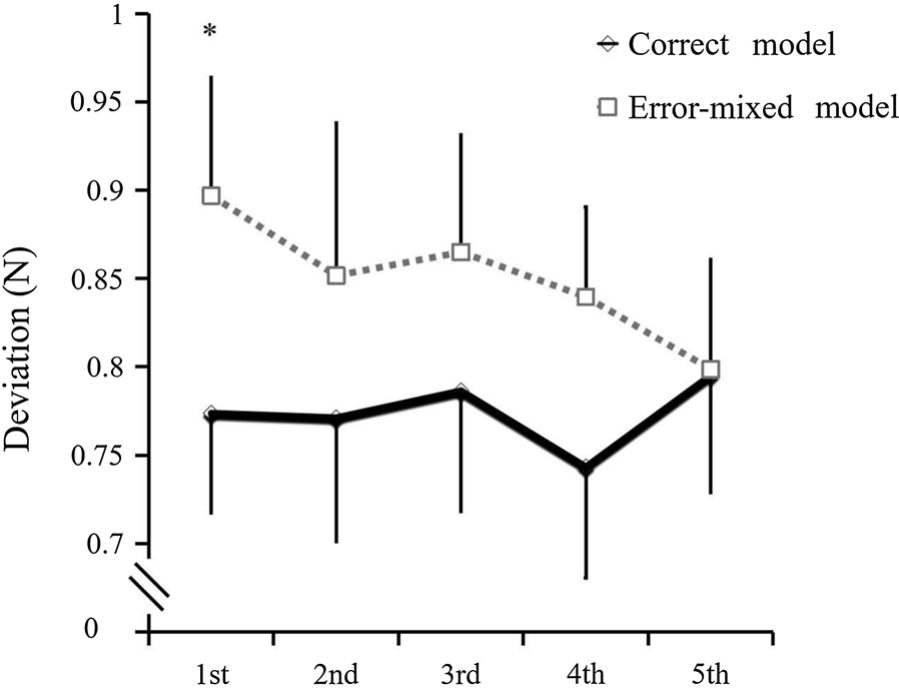
Learning process of Experiment 1. Changes in the deviation during the block’s sequence of the main training session. Error bars represent standardized error. *p < 0.05

Figure 4 shows the deviation changes of the five blocks in the main training sessions for both conditions. The deviations from the first to fifth block were 0.78 ± 0.04, 0.76 ± 0.06, 0.78 ± 0.05, 0.75 ± 0.05, and 0.79 ± 0.05 N in the correct condition, and 0.90 ± 0.06, 0.86 ± 0.08, 0.86 ± 0.06, 0.83 ± 0.05, and 0.78 ± 0.05 N the in error-mixed condition, respectively. The main effect of the condition was significant [F (1, 29) = 5.379, *p* = 0.028], which showed that the error-mixed condition had a higher deviation compared to the correct condition. With a post-hoc comparison, there was a significant difference between the two conditions in the first block (*p* = 0.041). There was no significant main effect of time [F (4, 116) = 0.493, *p* = 0.741] and no significant interaction [F (4, 116) = 0.951, *p* = 0.437] between the two conditions. There was no significant difference in the blocks within the error-mixed condition [F (4, 116) = 0.614, *p* = 0.653]. In addition, there was no significant difference in the deviation between the blocks of the correct condition [F (4, 116) = 0.337, *p* = 0.852].

### Experiment 2 (error and correct models)

**Fig. 5:**
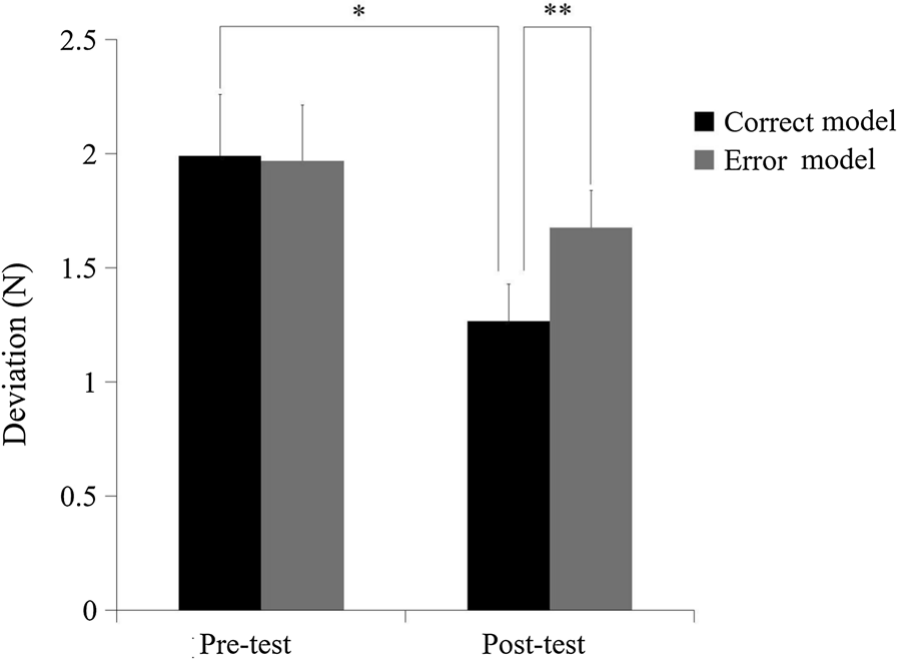
The deviation during the pre-test and post-test for each condition in Experiment 2. Error bars represent standardized error. *p < 0.05, **p < 0.01

The deviations of the two conditions (error and correct) and the two time levels (pre-test and post-test) are shown in Fig. 5. In the pre-test, no significant difference between the correct condition (1.99 ± 0.27 N) and error condition (1.97 ± 0.24 N) (*p* = 0.943) were found, while the deviation of the correct condition (1.27 ± 0.16 N) in the post-test was significantly lower compared to the error condition (1.68 ± 0.16 N) (*p* = 0.008). The main effect of time was significant [F (1, 19) = 6.43, *p* = 0.020]. The main effect of the condition was not significant [F (1, 19) = 1.54, *p* = 0.230]. No significant difference was found in the interaction between time and condition [F (1, 19) = 1.36, *p* = 0.259]. In multiple comparisons, there was no significant difference between the pre-and-post tests in the error condition (*p* = 0.212), whereas there was a significant difference between the pre-and-post-tests in the correct condition (*p* = 0.033).

**Fig. 6:**
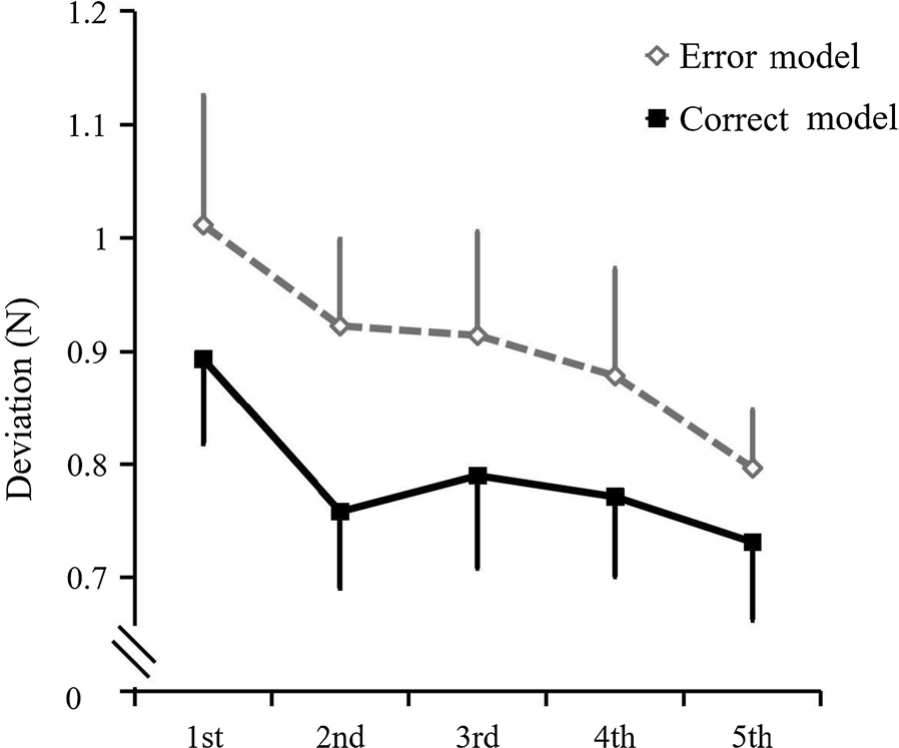
Learning process of Experiment 2. Changes in the deviation during the block’s sequence of the main training session. Error bars represent standardized error

Figure 6 shows the deviation changes during the blocks in the main training session for both conditions. The deviations from the first block to fifth were 0.89 ± 0.07, 0.76 ± 0.07, 0.79 ± 0.08, 0.77 ± 0.07, and 0.73 ± 0.07 N in the correct condition, and 1.01 ± 0.11, 0.92 ± 0.08, 0.91 ± 0.09, 0.87 ± 0.09, and 0.79 ± 0.05 N in the error condition, respectively. The main effect of the condition was significant [F (1, 19) = 7.597, *p* = 0.013], which showed that the error condition had a higher deviation compared to the correct condition. There was a significant main effect of time [F (4, 76) = 3.029, *p* = 0.023]. The interaction between the two conditions was not significant [F (4, 76) = 0.246, *p* = 0.911]. In multiple comparisons of the blocks within each condition, there was no significant difference among each block in the error-mixed condition [F (4, 76) = 1.954, *p* = 0.110]. There was also no significant difference within each block in the correct condition [F (4, 76) = 1.521, *p* = 0.205].

## Discussion

The results of Experiment 1 showed that the learning effect was better in the error-mixed condition. In Experiment 2, observing only error models had a higher deviation than the correct condition. These results showed that observing some error actions along with correct ones had an advantage in imitative learning, compared with observing only correct actions.

In Experiment 1, participants performed better when observing the error models along with correct actions compared to observing correct models only, which was partially consistent with previous studies. In a previous study, in imitative learning, viewing actions performed by expert and novice performers was better for motor learning compared to only viewing actions by experts. [12]. The novice model in the previous study was different from the error model in our study. In that, the novice model intended to achieve the goal, however the results did not achieve the goal since the poor process. In our study, the error model did not aim at the correct target at the beginning. Errors (4 N and 24 N) could certainly be judged as the output forces of the error actions were different from the target force (8 N). In other words, the models of 4 N or 24 N produced different goals compared to that of 8 N. We believe that the error information would be important for the learning process. Additionally, we used a pinching force generating task with a first-person perspective in which subjects map more closely onto the visual input of self-generated action to imitate synchronously [17]; this aspect differed from the previous study [12].

Some studies reported worse results from observing novice models than skilled ones [12, 24]. This could be because these studies were conducted with between-subject designs; the participants observed only the novice model and not the skilled (correct) model. Therefore, the participants might not have been able to ascertain those aspects of the observational action that were worse and those that were done well. As imitation has an automatic characteristic [25–27], it was possible that the participants imitated the worse aspects of the observational action if they did not distinguish the error. In Experiment 1, the participants first imaged the correct model by watching the correct models in the pre-training session. Then, in the main training session, since 50% of the models performed correct actions, the participants had a standard to discriminate the error easily. This view was confirmed in Experiment 2. In the main training session, when the correct model was not provided, the post-test showed decreased performance in the error model condition.

In Experiment 1, the deviation of the post-test showed that the performance was better in the error-mixed condition. However, the deviation of the main training session showed that it was worse in the error-mixed condition. This contrary result might cause some confusion regarding which condition was better for motor learning. However, motor learning is considered to produce relatively permanent changes in the capability for skilled behavior. The motor learning effect could be inferred from the performance, but it is not identical to the latter since it could easily have been affected by many other factors [28–30], such as, temporary facilitation or inhibition effects from the visual or auditory information, fatigue, and so on. As the setting of post-test was the same as between the two conditions and without any feedback or task-related visual information, we believe that the result of the post-test could more accurately reflect the learning effect. Additionally, the contrary result might be caused by the difference in execution conditions between the two conditions. This situation occurs as the correct action (congruent with the desired action) can facilitate action execution [31, 32]. In the error-mixed condition, the participants had less direct facilitation of correct observational actions, which might have led to worse temporary performance compared to the correct condition in the main training session.

We hypothesized that greater processing of task-related information occurred in the error-mixed condition, which may have boosted the learning effect. According to the goal-directed imitation theory, the imitator does not imitate the observed action wholly, but rather decomposes it into separate aspects [27, 33, 34]. These aspects are hierarchically ordered, and the useful aspect is utilized by the imitator [35]. When the participants observed error actions, they decomposed them into at least three aspects: error aspects (the thickness of the sponge and the velocity of pinching), correct aspects (the pinching direction and how to hold the pinch sensor), and meaningless aspects (the black background). The error aspects were utilized to detect whether the observer had the same errors. If the participant identified the same errors, these errors would be inhibited [27, 36]. This process could be considered to promote the detection of self-generated error, which is related to motor learning [20, 37, 38]. The correct aspects were used to imitate. In addition to the memory representation of the pinching action, the participants achieved the goal. It should be noted that Meaningless aspects would not be processed individually. When a participant observed the correct action, it would be decomposed into correct and meaningless aspects. The participants would not think further regarding what errors occurred in the correct action as they could identify the correct aspects from the instruction and the experience of the pre-training session. The correct aspects would be utilized to imitate and enhance the representation of the pinching action [39–41]. However, when the participants did not know the tasks well, they may not have distinguished between the correct aspects and errors.

In the error-mixed condition, the participants observed three types of actions, while in the correct condition, they observed only one. In other words, the participants had two error actions processes and one correct action process in the error-mixed condition, but only one correct action process in the correct condition. More processes and attention in the mixed condition might be the factor that promoted the learning effect [3, 12]. In the error-mixed model, participants were required pay attention to the vision. However, if all the action models were errors, subjects might have ignored the error only action owing to limited useful information from the vision. In the correct condition, subjects were “habituated” to the only correct models. Some neuroimaging research also indicated that the error-mixed condition had more processes compared to the correct condition. These previous studies showed that when a participant watched an error action, more cortical activation was found [42–44].

### Limitations

This study had several limitations. First, we only compared the immediate learning effect. Hence, it will be necessary to investigate the transfer and retention effects after 24 h or longer in the future. Second, we only assessed the force parameter and did not assess the kinematic parameters. Third, we only had behavioral data to confirm the processing of the errors made by others. Thus, a neuroimaging study is required. Finally, the mechanism and the process of the error-mixed condition remain unknown in the data obtained in our study.

## Conclusion

In this study, we found that the motor learning effect of observing a combination of error actions and correct actions was better than observing correct actions only in imitation learning. Observing error models only did not have an effect on imitation motor learning. These results indicate that the observation of errors made by others, in addition to observing correct models, could be used to improve the effect of imitation motor learning.

## Data Availability

The datasets used or analyzed during the current study are available from the corresponding author on reasonable request.
